# Allele frequency of a genetic risk variant for necrotizing meningoencephalitis in pug dogs from Europe and association with the clinical phenotype

**DOI:** 10.3389/fvets.2024.1407288

**Published:** 2024-05-22

**Authors:** Jana van Renen, Alexandra Kehl, Gesine Buhmann, Lara A. Matiasek, Yury Zablotski, Andrea Fischer

**Affiliations:** ^1^Small Animal Clinic, Centre for Clinical Veterinary Medicine, Ludwig-Maximilians-Universität München, Munich, Germany; ^2^Laboklin, Bad Kissingen, Germany; ^3^Comparative Experimental Pathology, School of Medicine, Technical University of Munich (TUM), Munich, Germany

**Keywords:** meningoencephalitis of unknown origin (MUO), immune-mediated encephalitis, genetic testing, autoimmune encephalitis, seizure, idiopathic epilepsy

## Abstract

**Introduction:**

Necrotizing meningoencephalitis (NME) in pugs is a potentially fatal disease, which needs lifelong treatment with immunosuppressive or immunomodulatory drugs and shares parallels with acute fulminating multiple sclerosis. Genetic variants of the DLA class II gene are associated with an increased risk for NME. Genetic testing is recommended prior to breeding. The aim of this study was to describe the current allele frequency of a previously identified NME risk variant in the European pug population. A secondary aim was to investigate the association of the NME risk variant with the clinical phenotype in pugs.

**Methods:**

Results of genetic testing for the CFA12:2605517delC variant in European pugs between 2012 and 2020 were retrieved (*n* = 5,974). A validated questionnaire was mailed to all submitters of samples for further information on neurological signs, diagnostic tests, and disease course.

**Results:**

The allele frequency of the CFA12 NME risk variant was 25.7% in the European pug population dogs; 7.4% of the dogs were homozygous and 36.7% were heterozygous for the NME risk variant on CFA12. Completed questionnaires were available in 203 dogs including 25 dogs with epileptic seizures or other neurological signs. The clinical phenotype was consistent with NME in 3.9% with a median age of onset of 1.0 years, and indicative of idiopathic epilepsy in 2.9% with a median onset of 2.5 years. Eleven dogs remained unclassified. Pugs with the NME phenotype were significantly more frequently homozygous for the NME risk variant on CFA12 compared to pugs ≥6 years without neurological signs or seizures (*p* = 0.008).

**Discussion:**

The CFA12:2605517delC genetic risk variant is widely distributed in the European pug population and frequently homozygous in pugs with a NME phenotype. The data support the clinical relevance of the CFA12:2605517delC genetic risk variant.

## 1 Introduction

Pugs are a popular dog breed all around the world. However, neurological disorders such as vertebral malformations (1, 2), arachnoid diverticula (3, 4), congenital hydrocephalus (5, 6), brain tumors (7, 8), and necrotizing meningoencephalitis (NME) (9–11) are common in this breed. NME is a subgroup of meningoencephalitis of unknown origin (MUO), a disease describing an idiopathic, non-infectious and suspected immune-mediated central nervous system inflammation; subgroups of MUO are granulomatous meningoencephalitis (GME) and necrotizing encephalitis [further divided into NME and necrotizing leukoencephalitis (NLE)] (9–11). The first description of NME in pugs was 1989 in California (12). Years ago, NME was considered a breed-specific disease only occurring in pugs. Nowadays this disease is known to occur in many other breeds, mainly small breeds (9), including Chihuahua (13), Maltese dog (14, 15), Yorkshire terrier (16–19), French bulldog (20), Pekingese dog (21), West Highland White Terrier (22), Papillon, Shih Tzu, Coton de Tulear, and Brussels Griffon (23). NME is a potentially fatal, rapidly progressive brain disorder; reported survival times from time of diagnosis to death are often only a few days or months (9, 11, 23, 24). The etiology of NME still remains unknown: different theories have been discussed in the literature and the definitive diagnosis relies on histopathological examination and requires biopsy or post mortem examination of the brain (9–11, 25). A multifocal etiology is suspected, most likely involving a combination of genetic predisposition and trigger factors (environmental and infectious agents) (10, 11, 26–29). A presumptive diagnosis is based on signalment, neurological presentation and diagnostic findings on brain imaging [magnetic resonance imaging (MRI), computer tomography (CT)] and cerebrospinal fluid (CSF) analysis (9–11, 26, 30). The median age at onset of NME in pugs is 18 months. Female dogs appear predisposed and many dogs are < 4 years old at disease onset (9, 10, 19, 24, 31, 32). Common neurological signs of NME are forebrain signs such as seizures, central blindness, visual deficits and abnormal behavior due to most severe lesions within the cerebral cortex (9, 10). Other common signs are circling, reduced consciousness, ataxia, head pressing and cervical hyperesthesia (9, 10, 22, 32–34). Typical histopathologic features of NME include non-suppurative inflammation of meninges, cerebral cortex and subcortical and deep white matter (9, 23). The main histological finding is multifocal necrosis in different manifestations depending on the stage of the disease: neuronal necrosis and gliosis can progress to parenchymal cavities (10). There is a loss of demarcation between gray and white matter and marked infiltration of mixed mononuclear cells (plasma cells, lymphocytes, and histiocytes) in the cerebral hemispheres and meninges (9, 10, 34). The most severe lesions are found in the leptomeninges, cerebral cortex, corona radiata, and subcortical white matter; rarely lesions are also found within the brainstem and cerebellum (10). Geer et al. identified a genetic risk variant for NME in pugs; they found a strong singular association with the DLA class II genes (28). A German diagnostic laboratory offers a genetic test for NME in pugs which is based on the CFA12:2605517delC variant on the DLA-DPB1 gene as Corneveaux et al. describes (35). The aim of the present investigation was to describe the allele frequency of the NME risk variant in the European pug dog population. A secondary aim was to investigate whether the variant was associated with a particular clinical phenotype.

## 2 Materials and methods

An allele frequency study and a questionnaire study were performed in cooperation with an international veterinary diagnostic laboratory (Laboklin GmbH, Bad Kissingen, Germany) with ethical permission (no. 147-16-10-2018, LMU Munich).

### 2.1 Allele frequency study

The database of the laboratory was reviewed for pugs tested for the CFA12:2605517delC gene variant (2012–2020). Basic information regarding the genetic test result, date, sex, and age of the dog when genetic testing was performed were retrieved. The genetic test results were routinely reported as NME/NME (homozygous on both alleles for the CFA12:2605517delC gene variant), WT/NME (heterozygous, carrier) and WT/WT (wildtype on both alleles). The period allele frequency of the NME risk variant in the central European pug population was calculated as [(2xNME/NME + 1xWT/NME)/2xall tested dogs] × 100.

### 2.2 Questionnaire design

A standardized online questionnaire was designed using the online application Microsoft Forms in German and English language. The survey included 53 questions: 34 single choice questions, 14 free text questions, and five multiple choice questions. The number of questions varied (12–49 questions), depending on the clinical signs of the dog. The questions focused on demographic information and presence of neurological signs including epileptic seizures in tested pugs as outlined below.

#### 2.2.1 Requested demographic information

The survey asked for sex, date of birth, date of death (for dogs that were no longer alive), cause of death, and the time since the last contact with the pet owner. Furthermore, the survey asked whether a veterinarian, breeder, or the caregiver of the dog answered the questionnaire.

### 2.3 Description of the clinical phenotype

The survey asked whether the dog had ever experienced seizures or other neurological signs. Additional questions were asked to obtain more detailed descriptions of the phenotype. In case of seizures: age at onset, whether recurrent seizures occurred consistent with a diagnosis of epilepsy, seizure type, seizure frequency, whether cluster seizures or status epilepticus occurred, whether treatment with antiseizure medication was started and duration of treatment. In case of other neurological signs: age at onset, details on neurological signs (ataxia, paresis, cranial nerve signs, and mental status) and whether a specific underlying cause could be identified. Diagnostic test results: whether blood tests, imaging of the brain or spinal cord, e.g., MRI, CT, and/or CSF were carried out and whether the tests were normal or abnormal. Diagnosis and treatment: The participants were asked whether a final clinical diagnosis could be obtained. The treatment and outcome were reviewed.

### 2.4 Distribution of the questionnaire

All customers who had submitted a sample (EDTA blood or saliva) from a pug for diagnostic testing for the CFA12:2605517delC variant on the DLA-DPB1 gene between 2012 and 2020 were contacted by the laboratory and invited per e-mail to complete an online questionnaire. In the e-mail, the submitters were provided with information about the dog from which the genetic test was initiated (name of the dog, name of the caregiver, date of birth of the dog, sex of the dog, reference number of the test, and date of the test) and a link to an online questionnaire. The submitters of the samples were also asked to forward the e-mail and questionnaire link to the current caregiver of the pet. The submitters could provide contact details for additional questions. The survey was performed according to the General Data Protection Regulation (GDPR) and with permission of the data protection officer of Laboklin GmbH and LMU Munich. The results of the survey were exported as an excel file and anonymously transmitted to the study investigators. All data were reviewed. Cases were excluded if the questionnaire was not completed.

### 2.5 Clinical phenotype

The diagnostic labels “suspected NME” and “suspected idiopathic epilepsy (IE),” or “undefined neurological disease” were assigned to each pug with seizures or other neurological signs (36) according to predefined criteria (Table 1). For dogs without seizures or neurological signs the diagnostic label “no neurological signs or seizures” was assigned for pugs which were 6 years or older at the time of the questionnaire because younger dogs could still develop NME or IE.

**Table 1:** Criteria for diagnostic labels.

Suspected idiopathic epilepsy	Tier I	Age at onset 6 months−6 years
		Two or more epileptic seizures, no other neurological signs
		Unremarkable blood examination
		Minimum follow-up 1 year after first seizure without progression to other neurological signs
	Tier II	Same criteria as IE tier I
		Unremarkable brain MRI
Suspected NME	Tier I	Age at onset < 7 years
		Epileptic seizures or other neurological signs, alone or in combination
		Progressive clinical course
	Tier II	Characteristic abnormalities in MRI or CT (parenchymal changes) or CSF pleocytosis
		Otherwise, same criteria as NME (tier I)

### 2.6 Statistics

Risk allele frequency and 95% confidence intervals for the CFA12:2605517delC variant on the DLA-DPB1 gene were calculated. Relative risk (OR, odds ratio) for IE and NME based on genetic test results was calculated via multivariable logistic regression. Associations between sex and genetic test results were assessed with Chi Square test. Only cases with information on clinical follow-up were considered for risk calculations. All calculations and statistical analysis were conducted using R statistical software (R version 4.2.1; 2022-06-23) and Microsoft Excel^®^ (Microsoft Office 2016).

## 3 Results

Within a 9-year period (2012–2020), 6,135 genetic tests for the CFA12:2605517delC variant on the DLA-DPB1 gene were requested in pugs; 161 submitted incorrect material for genetic testing. Thus, 5,974 valid tests were performed. DNA was retrieved from EDTA blood or saliva samples. The samples originated from 3,443 female (57.6%) and 2,243 male dogs (37.5%) from 28 different countries: Germany, England, Estonia, Austria, Switzerland, Italy, Latvia, Lithuania, Netherlands, Norway, Croatia, Poland, Ukraine, Spain, Slovakia, Sweden, Slovenia, Russia, Romania, Serbia, Island, Hungary, Greek, French, Denmark, Czechia, Bulgaria, and Belarus.

### 3.1 Allele frequency of the CFA12 risk variant in pugs in Europe

The risk allele frequency of the CFA12:2605517delC variant on the DLA-DPB1 gene was 25.7%. 7.4% of the pugs were homozygous for the CFA12 risk variant (NME/NME; 444/5974), 36.7% were heterozygous (WT/NME; 2190/5974) and 55.9% were wildtype on both alleles (WT/WT; 3340/5974; Table 2). There was no significant association between the sex of the dogs and the genetic test results (*p* = 0.24).

**Table 2:** Genotypes and disease associations.

	**No. dogs**	**NME/NME**	**WT/NME**	**WT/WT**
Study population	5,974 dogs	7.4% (444/5,974)	36.7% (2,190/5,974)	55.9% (3,340/5,974)
Questionnaire cohort	203 dogs	13.8% (28/203)	31.5% (64/203)	54.7% (111/203)
Suspected NME	8 dogs	3% (6/203)	0.5% (1/203)	0.5% (1/203)
Suspected IE	6 dogs	0%	2.5% (5/203)	0.5% (1/203)
Undefined neurological disease	11 dogs	1.5% (3/203)	1.5% (3/203)	2.5% (5/203)
Dogs without neurological signs or seizures	178 dogs	9.4% (19/203)	27.1% (55/203)	51.2% (104/203)
< 6 years	119 dogs	4.9% (10/203)	18.2% (37/203)	35.5% (72/203)
≥6 years	59 dogs	4.4% (9/203)	8.9% (18/203)	15.8% (32/203)

### 3.2 Questionnaire cohort (*n* = 203)

**Figure 1:**
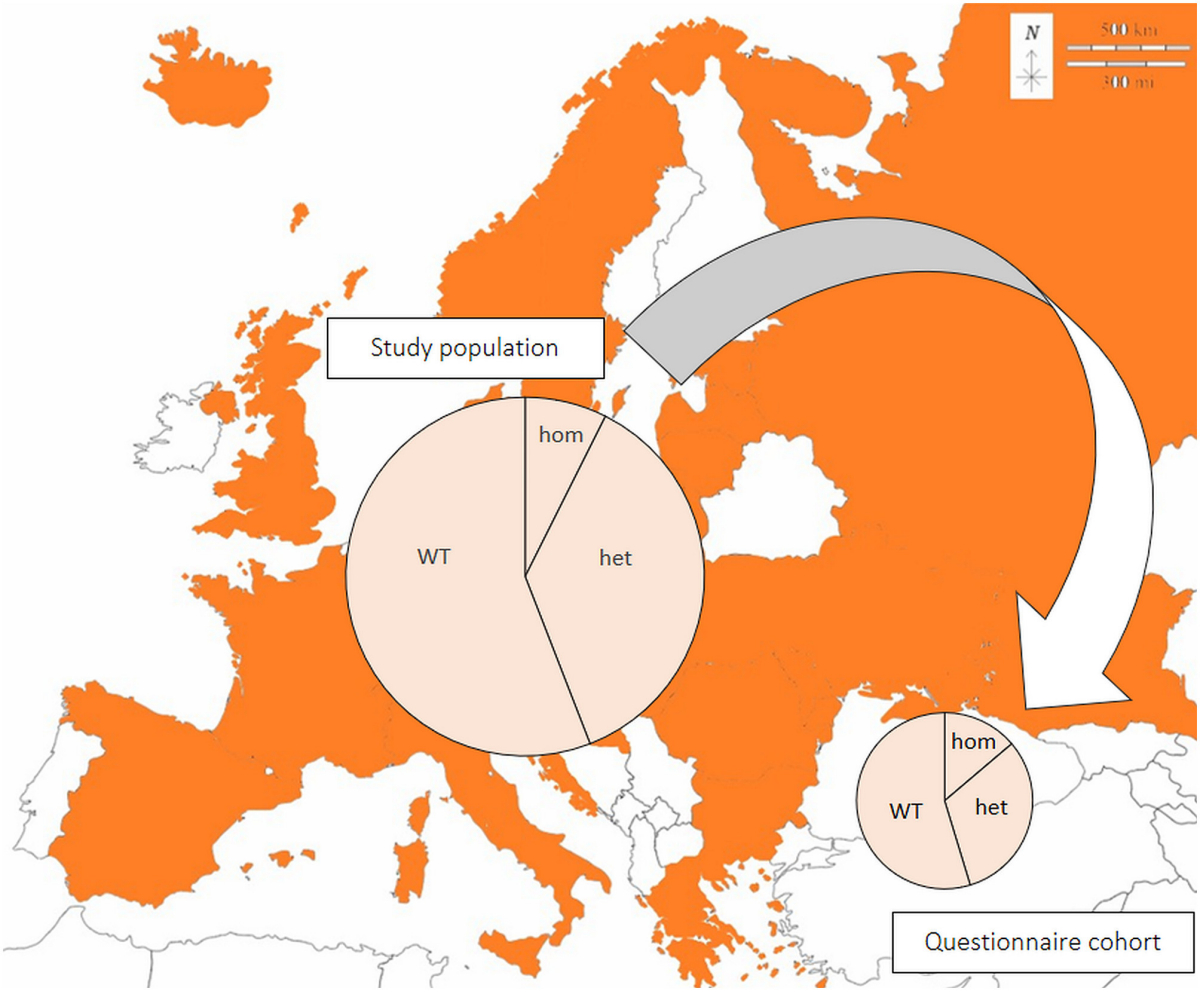
Genetic test results in the European pug study population (*n* = 5,974) and a representative questionnaire cohort (*n* = 203). Samples originated from all colored countries. Large circle: all samples (*n* = 5,974); small circle: questionnaire cohort (*n* = 203); hom: homozygous, NME/NME; het: heterozygous, WT/NME; WT: wildtype on both alleles, WT/WT. Modified from: https://d-maps.com/pays.php?num_pay=192&lang=de.

In total, 213 questionnaires (161 German, 52 English) were submitted (Figure 1). Ten questionnaires were incomplete and excluded, thus 203 valid data sets were available for evaluation. Questionnaires were submitted by the breeders (49.3%; 100/203), veterinarians (29.6%; 60/203), or pet owners (20.7%; 42/203). The median age of dogs at the time of genetic testing was 12 months (range 0–143 months; mean 19.4 months). 59.1% of the dogs (120/203) were female, and 39.4% (80/203) male. Sex was not specified in three dogs. The genetic test result was NME/NME in 13.8% (28/203), WT/NME in 31.5% (64/203), and WT/WT in 54.7% (111/203). Sex distribution and genetic test results were similar in the study population and the questionnaire cohort (Table 2).

At the time of completing the questionnaire study, 8.4% (17/203) of the dogs were no longer alive. The mean age at death was 60.2 months (range: 4–144 months; median: 50 months). Ten dogs had died or were euthanized due to neurological signs or epileptic seizures and seven dogs (7/17) due to other causes (torsion of the lung, pancreatitis, mammary gland tumor with pulmonary metastasis, pancreatitis, anaphylactic reaction to a bee sting, undefined collapsing episode, pancreatic tumor, or hit by car). The submitters of the questionnaires had seen the dog or contacted the pet owner < 3 months ago (150 dogs), 3–6 months ago (17 dogs), 6–12 months ago (11 dogs), 12–24 months ago (10 dogs), and more than 24 months ago (15 dogs).

### 3.3 Clinical phenotype

Overall, 12.3% of the dogs of the questionnaire cohort (25/203) showed epileptic seizures or neurological signs and 87.7% (178/203) had no neurological signs or seizures (119 dogs < 6 years, 59 dogs ≥6 years).

#### 3.3.1 Suspected NME

**Figure 2:**
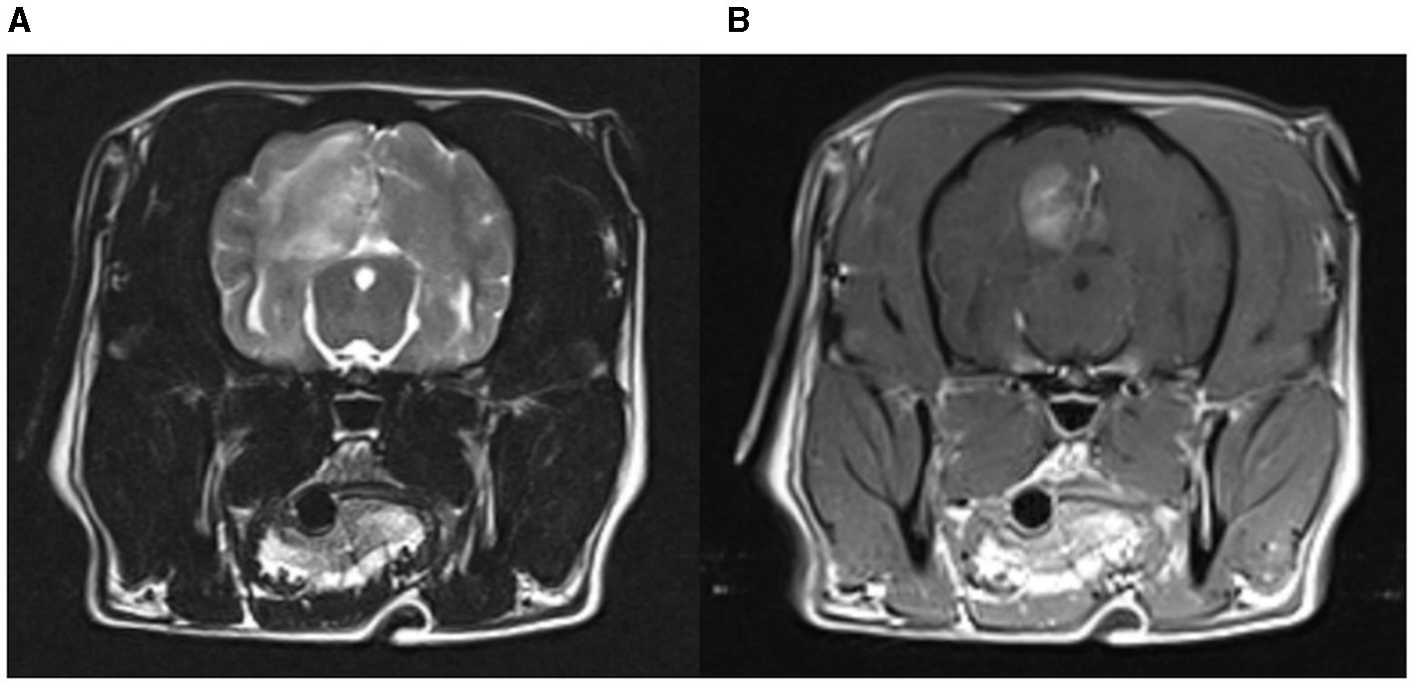
MRI of a pug with NME. An extensive lesion with heterogenous T2W hyperintensity in the left cerebral hemisphere. Contrast enhancement is most distinct in the center of the lesion at the gray-white matter border. **(A)** T2-weighted brain MRI (transverse plane); **(B)** T1-weighted brain MRI with contrast (transverse plane).

NME was considered in 3.9% (8/203) of the pugs of the questionnaire cohort (1 dog tier I, 7 dogs tier II). The genetic test result was NME/NME in 75% (6/8), WT/NME in 12.5% (1/8) and WT/WT in 12.5% (1/8). 62.5% (5/8) were male, 25% (2/8) female, and in one dog the sex was unknown. Six of the pugs showed epileptic seizures with (three dogs) or without other neurological signs (three dogs) and two pugs showed only neurological signs. Median age at onset of neurological signs was 1 year (range: < 1–6 years). Blood examination was performed in 7 dogs (7/8; 87.5%) and showed mild changes in two dogs (mild anemia, mildly elevated creatinine). MRI was performed in 5 dogs (5/8; 62.5%) and CT in one dogs and parenchymal changes indicative of NME was reported in each of the dogs (Figure 2). CSF was evaluated in six dogs (6/8; 75%) and abnormal findings were indicated in five dogs. Altogether, in five dogs brain imaging together with CSF sampling was performed, in one dog only brain imaging and in one dog only CSF was performed. Most dogs (7/8; 87.5%) were treated for suspected NME; in one dog the treatment was unknown. Treatment consisted of prednisolone (unknown dosages), antibiotics, omeprazole, lansoprazole, and lomustine. Treatment success was described as follows: After initiation of treatment, one dog did not show any more neurological signs, three dogs continued to show mild signs, in one dog the neurological status did not change, and one dog showed worsening of neurological signs. 87.5% (7/8) of the dogs with suspected NME died or were euthanized and one dog was lost to follow up. Death was attributed to neurological disease in all dogs but one: four dogs neurological signs other than seizures, two dogs epileptic seizures, and one dog mammary gland tumor. The median age at death was 50 months (range: 9–83 months).

#### 3.3.2 Suspected IE

Age of onset and disease course were consistent with IE in 2.9% (6/203) of the pugs of the questionnaire cohort (4 tier 1, 2 tier 2). The genetic test result was WT/NME in 83.3% (5/6) and WT/WT in 16.7% (1/6). Females and males were equally affected. All the dogs had a history of seizures for at least 1 year or longer (range 1–7 years) without any progression to other neurological signs. The pugs were between 1 and 5 years old (median 2.5 years) at the time of the first seizure. Laboratory examination (hematology, serum biochemistry) was performed in each dog. Abnormal results were elevation of postprandial serum bile acids in one dog. MRI was performed in two of the dogs and in one of them CSF was also evaluated, all with unremarkable results. Five pugs with suspected IE were treated with anti-seizure medication (ASM) for a minimum of 1 year or longer (two dogs 2 years, one dog 4 years). One dog remained drug naive. All pugs with suspected IE were still alive and aged between 3 and 11 years at the time of study conclusion.

#### 3.3.3 Undefined neurological disease

5.4% (11/203) of the dogs showed neurological signs or seizures, which could not be attributed to NME or IE because inclusion criteria were not fulfilled: three only epileptic seizures, three neurological signs with additional seizures, and five only neurological signs without seizures. Hydrocephalus was diagnosed as the underlying cause in one dog; in the other dogs the cause of the neurological disease remained undefined.

### 3.4 Genetic risk

Dogs with suspected NME were significantly more likely homozygous for the CFA12:2605517delC variant on the DLA-DPB1 gene than dogs ≥6 years with no neurological signs or seizures (*p* = 0.008).

## 4 Discussion

This study described the genetic test results of 6,135 European pugs tested for the CFA12:2605517delC variant on the DLA-DPB1 gene. The results demonstrated a surprisingly high allele frequency of the gene variant in the European pug population (25.7%), with 7.4% of the European pugs at high risk and 13.8% of the questionnaire cohort at high risk, as defined previously (37), but stays in contrast to other studies (38). Questionnaire data on the neurological signs and disease course of a representative subgroup indicate that the genetic risk variant is associated with an NME phenotype.

The high allele frequency of the CFA12:2605517delC gene variant is a considerable problem for the breeding population considering the association with an NME phenotype. Several genetic risk loci are described for NME: one risk locus on chromosome 12 within the dog leukocyte antigen (DLA) major histocompatibility (MHC) II complex (38–40), and another risk locus on chromosome 15 in the pug and chromosome 4 in the maltese dog (14). It has been stated that genetic risk variants are not suitable as diagnostic tests for NME because not all homozygous dogs may develop the disease (28, 39). In this study, 19 dogs with a homozygous NME/NME genetic test result did not show neurological signs or seizures; however, 10 of them were still younger than 6 years at the time of the questionnaire. It is possible that these pugs could still develop an NME, or had only a subclinical phenotype of NME as proposed by others (37). The further clinical course of these dogs was unknown. These risk loci place homozygous pugs only at high risk to develop NME, and there is a need for further longitudinal studies. A causal genetic variant with monogenic inheritance has yet not been identified for NME.

Furthermore, it is currently unknown to which degree these risk variants in the canine leukocyte antigen DLA MHC II complex are present in other breeds suffering from NME. The association between the MHC class II proteins and disease development is also known in humans with multiple sclerosis (MS) (28). A recent study described a potential early clinical phenotype of NME in asymptomatic but genetically at risk pugs, which might suggest the need to start early diagnosis and therapy for these dogs even in the absence of neurological signs (37, 41).

The present investigation demonstrated an allele frequency of the risk variant of 25.7% in the European study population. These results are similar to a recent North American investigation (37). The results demonstrated also that dogs with a clinical NME phenotype were significantly more likely homozygous for the NME risk variant on CFA12 than dogs ≥6 years without neurological signs (*p* = 0.008) thus providing further support for the pathogenic potential of the variant or the associated haplotype. This association was only seen for pugs with a phenotype reminiscent of NME but not for pugs with IE or undefined neurologic disease. Therefore, the data support the causality of the tested CFA12 risk variant and suggest that the genetic test could serve as a useful test for breeders to reduce the allele frequency of NME in pugs (25).

Median age of onset was 1 year in dogs from the questionnaire cohort with suspected NME. This is consistent with previous reported age ranges in NME (9, 10, 19, 24, 31, 32). The data did not replicate the previously reported female sex preference; as 62.5% of the dogs with suspected NME were male, but numbers are too low for valid conclusions. Six (75%) of the dogs in this group had a NME/NME genetic test result and thus were homozygous for the CFA12 risk variant and at high risk for NME. These dogs had neurological signs indicative of forebrain disease with seizures, visual disturbances, blindness and aimless walking (9, 10, 21, 26). Typically MRI lesions in NME are described as multifocal, asymmetrical, cerebral T2W- and FLAIR-hyperintense, T1W-hypointense signal change, affecting the cortical gray and subcortical white matter as well as contrast enhancement, midline shift, mass effect, and loss of white and gray matter demarcation (10, 12, 42, 43). Generally there is a lymphomonocytic pleocytosis and elevation of protein (9, 10, 43, 44). The dogs with suspected NME from the questionnaire cohort also showed multiple T2W- and FLAIR- hyperintense signal changes in the cerebrum in four dogs, contrast enhancement in five dogs and midline shift in four dogs. Brain imaging with CT was performed in one dog and showed signs of mass effect, midline shift and diffuse contrast enhancement of the cerebrum. The cerebrospinal fluid analysis revealed increased CSF protein concentrations in three dogs, a lymphohistiocytic pleocytosis in four dogs and was normal in one dog.

There are many different treatment options for NME, which is considered a subgroup of MUO. Most protocols use prednisolone as the baseline immunosuppressive agent and other immunosuppressive medications can be added, most commonly cytosine arabinoside, but cyclosporine, lomustine, and others may also be used (9, 10, 43, 45–51). Another therapeutic option is radiotherapy (9, 52, 53). Five of the eight dogs in our cohort were treated with prednisolone and one dog with a combination of prednisolone and lomustine, but dosages were not reported. None of the dogs with suspected NME was still alive at the time of the questionnaire, which further supported the conclusion that NME was the underlying disorder as this disease is frequently associated with a fatal disease course. However, a definite diagnosis would require at least review of the MRI or confirmation with a post-mortem examination.

Up to now pugs are not considered predisposed breeds for idiopathic epilepsy. It could be difficult to differentiate idiopathic epilepsy from NME without MRI and CSF analysis because epileptic seizures could occur in both disorders, and the onset of NME and IE is in the same age range. Idiopathic epilepsy was suspected in six dogs from the questionnaire cohort, but the supporting evidence was limited because only two dogs had an MRI exam (tier 2 confidence level as defined by the International Veterinary Epilepsy Task Force consensus statement) (36). The fact that all dogs with suspected IE were still alive at study conclusion, had long survival times and no other neurological signs provides further support for the presence of idiopathic epilepsy in these pugs. None of the dogs with suspected IE was at risk for NME (homozygous), but five out of six dogs showed a low risk for NME (heterozygous). As the MRIs were unavailable for review, we cannot exclude subtle MRI changes in heterozygous dogs as proposed in a recent study (37).

There have been interesting research efforts on the pathologic features, potential biomarkers and the mechanisms and triggers for disease development considering the parallels to multiple sclerosis or autoimmune encephalitis in humans (25, 54, 55). Various infectious agents, environmental and genetic factors are considered as contributing factors for NME, but so far no trigger agent could be found, and a single sensitive and specific ante-mortem test is yet not available (25, 38, 56–60). Previously some authors focused on glial fibrillary acidic protein (GFAP) and GFAP autoantibodies in dogs with MUO and NME as a marker for astrocyte activity following CNS-injury, stress or dysfunction (61). GFAP and GFAP autoantibodies in CSF could possibly serve as a unique marker of NME in pug dogs (62–65). Other potentially useful biomarkers in differentiating the different pathologic subtypes of MUO could be cytokine and chemokine mRNA and protein expression in brain tissue; markedly higher expression of IFN-g are described in NME lesions than in GME/NLE lesions as well as an increased IL-17 expression in GME lesions (66). At present the trend is to focus less on the pathologic differences between GME, NME and NLE, rather these are considered to represent autoimmune encephalitis with overlapping phenotypes with parallels to MS. The emerging question is the recognition of biomarkers (clinical scores, imaging findings, serum biomarker) for the prediction of the clinical course (41, 67, 68).

Our study had several limitations. The answers were from groups of people (veterinarians, breeders, and owners) with different experience, so the ability to perform a neurological examination and to detect slight abnormalities in the behavior of the dog could vary widely. Furthermore, we received only limited data about the treatment and the further course of the disease from the initial diagnosis until death. We were unable to review the MRI and results of CSF analysis in detail, and in many dogs with presumptive diagnosis of IE only CT was performed. We cannot rule out the potential for bias. It is possible that dogs with a higher genetic risk and neurological signs participated preferentially in the questionnaire study.

## 5 Conclusion

We describe the results of genetic testing for a CFA12 variant in a large population of European Pug dogs; the CFA12:2605517delC risk variant is widely distributed in the European pug dog population and pugs with an NME phenotype are frequently homozygous for the tested CFA12 risk variant. These data demonstrate the clinical relevance of the risk variant and open avenues for future longitudinal studies of pugs at risk.

## Data availability statement

The original contributions presented in the study are included in the article/supplementary material, further inquiries can be directed to the corresponding author.

## Ethics statement

The animal study was approved by Ethics Committee of the Veterinary Faculty LMU Munich, Germany. The study was conducted in accordance with the local legislation and institutional requirements.

## Author contributions

JvR: Conceptualization, Data curation, Investigation, Methodology, Visualization, Writing – original draft. AK: Data curation, Investigation, Supervision, Writing – review & editing. GB: Data curation, Writing – review & editing. LM: Writing – review & editing. YZ: Methodology, Software, Writing – review & editing. AF: Conceptualization, Data curation, Supervision, Writing – review & editing.
